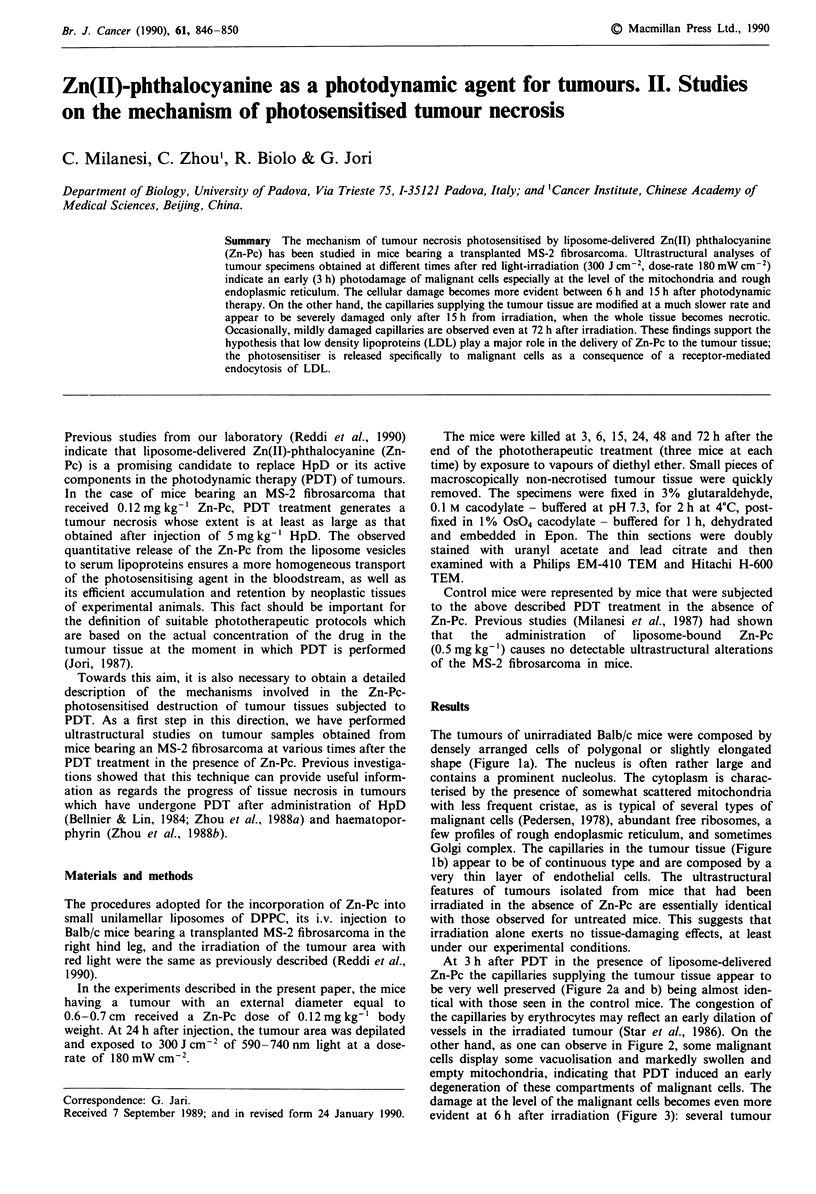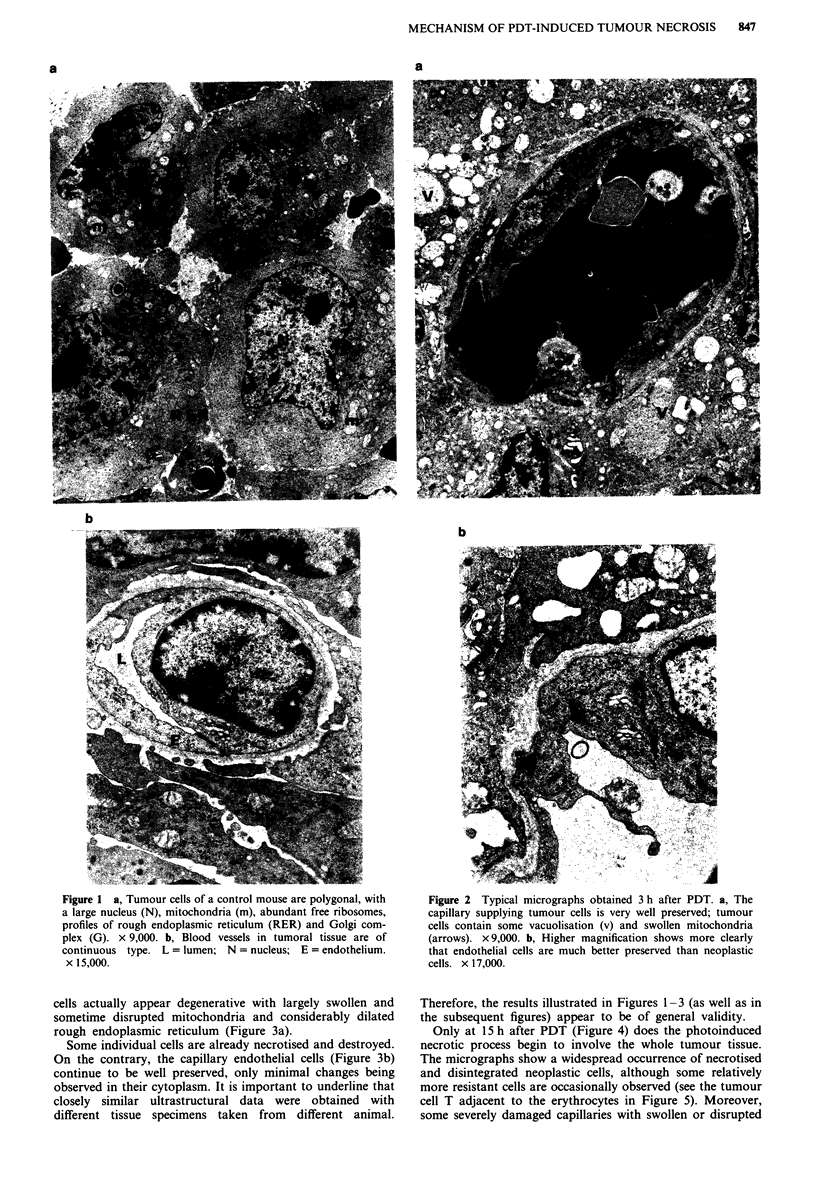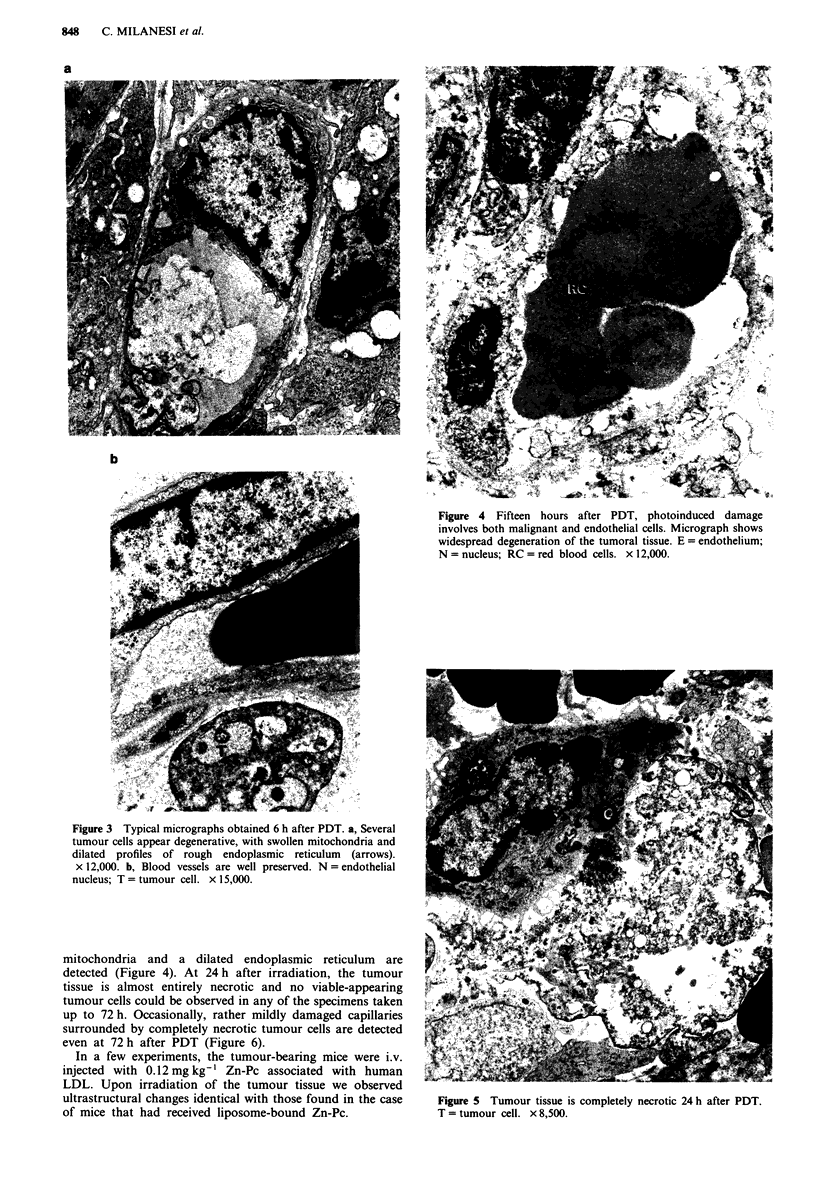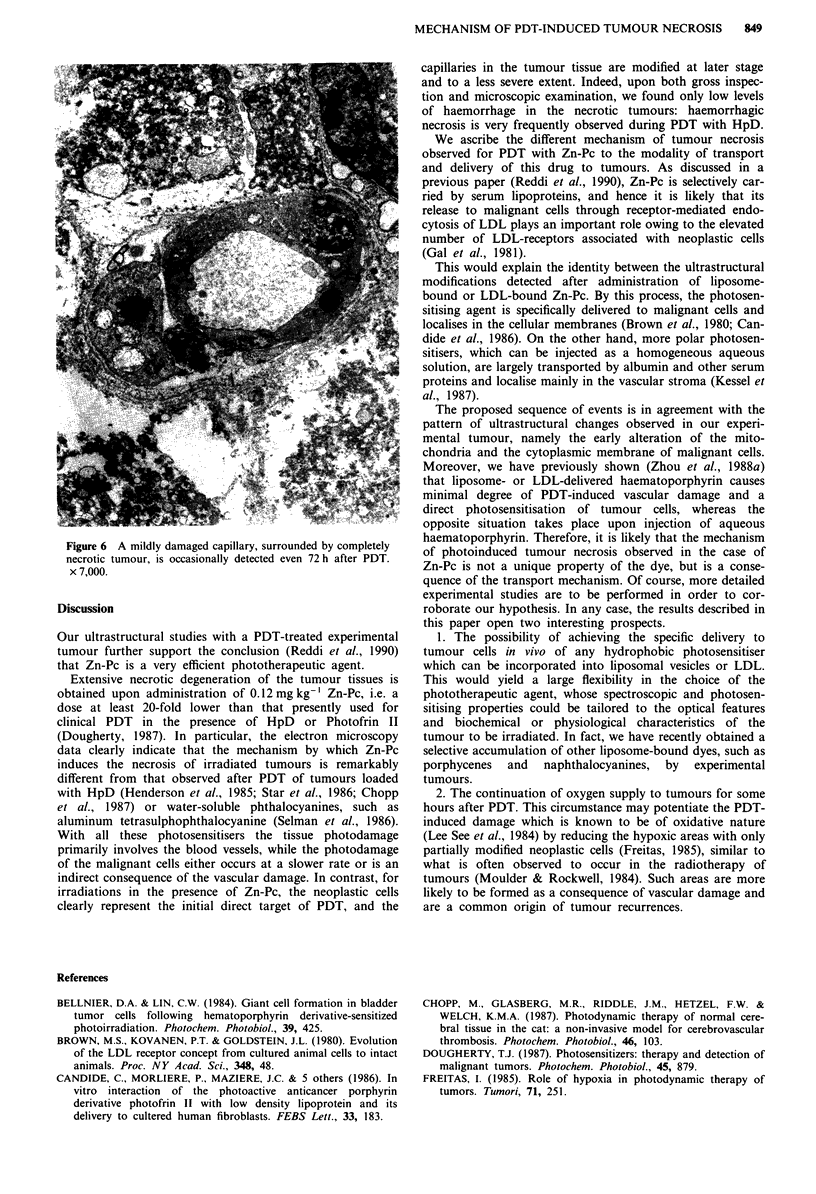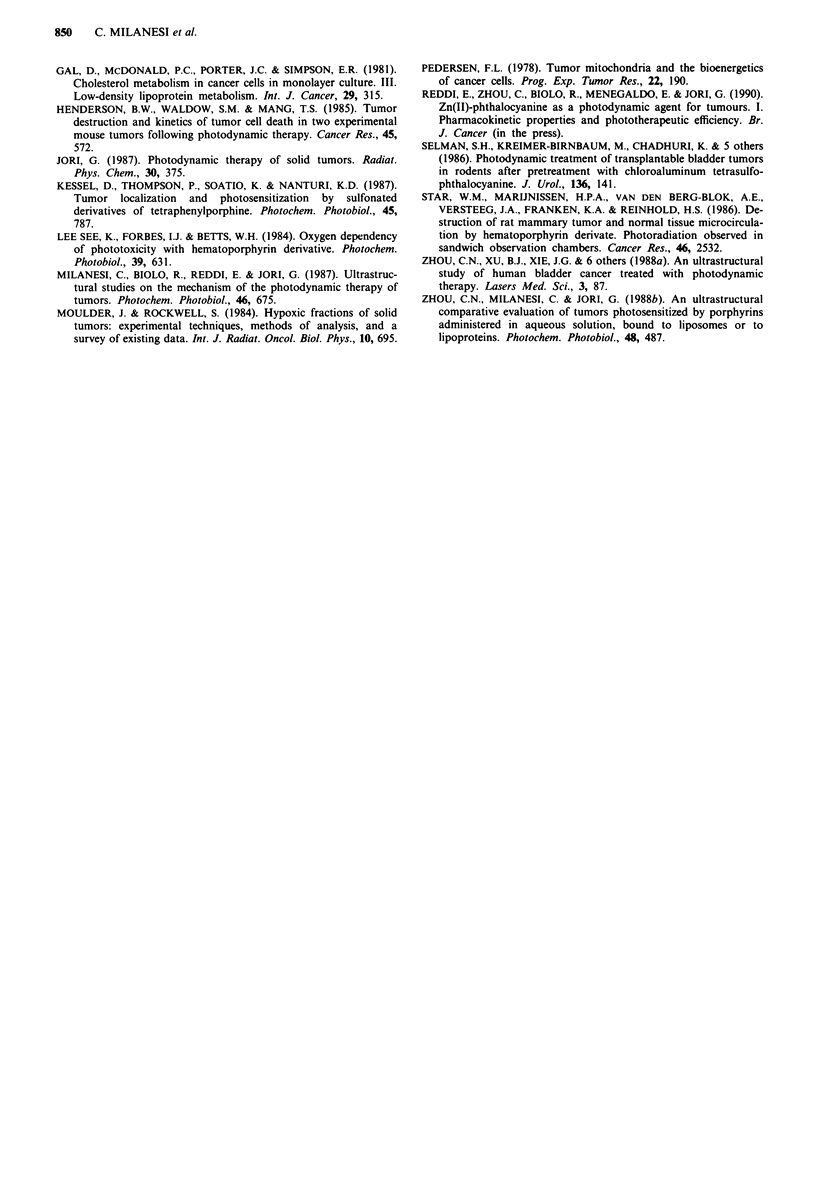# Zn(II)-phthalocyanine as a photodynamic agent for tumours. II. Studies on the mechanism of photosensitised tumour necrosis.

**DOI:** 10.1038/bjc.1990.189

**Published:** 1990-06

**Authors:** C. Milanesi, C. Zhou, R. Biolo, G. Jori

**Affiliations:** Department of Biology, University of Padova, Italy.

## Abstract

**Images:**


Br. J. Cancer (1990), 61, 846-850 © Macmillan Press Ltd., 1990

### Zn(II)-phthalocyanine as a photodynamic agent for tumours. II. Studies on the mechanism of photosensitised tumour necrosis

C. Milanesi, C. Zhou[1], R. Biolo & G. Jori

*Department of Biology, University of Padova, Via Trieste 75, I-35121 Padova, Italy; and [1]Cancer Institute, Chinese Academy of Medical Sciences, Beijing, China.*

**Summary** The mechanism of tumour necrosis photosensitised by liposome-delivered Zn(II) phthalocyanine (Zn-Pc) has been studied in mice bearing a transplanted MS-2 fibrosarcoma. Ultrastructural analyses of tumour specimens obtained at different times after red light-irradiation (300 J $cm^{-2}$, dose-rate 180 mW $cm^{-2}$) indicate an early (3 h) photodamage of malignant cells especially at the level of the mitochondria and rough endoplasmic reticulum. The cellular damage becomes more evident between 6 h and 15 h after photodynamic therapy. On the other hand, the capillaries supplying the tumour tissue are modified at a much slower rate and appear to be severely damaged only after 15 h from irradiation, when the whole tissue becomes necrotic. Occasionally, mildly damaged capillaries are observed even at 72 h after irradiation. These findings support the hypothesis that low density lipoproteins (LDL) play a major role in the delivery of Zn-Pc to the tumour tissue; the photosensitiser is released specifically to malignant cells as a consequence of a receptor-mediated endocytosis of LDL.

Previous studies from our laboratory (Reddi *et al.*, 1990) indicate that liposome-delivered Zn(II)-phthalocyanine (Zn-Pc) is a promising candidate to replace HpD or its active components in the photodynamic therapy (PDT) of tumours. In the case of mice bearing an MS-2 fibrosarcoma that received 0.12 mg $kg^{-1}$ Zn-Pc, PDT treatment generates a tumour necrosis whose extent is at least as large as that obtained after injection of 5 mg $kg^{-1}$ HpD. The observed quantitative release of the Zn-Pc from the liposome vesicles to serum lipoproteins ensures a more homogeneous transport of the photosensitising agent in the bloodstream, as well as its efficient accumulation and retention by neoplastic tissues of experimental animals. This fact should be important for the definition of suitable phototherapeutic protocols which are based on the actual concentration of the drug in the tumour tissue at the moment in which PDT is performed (Jori, 1987).

Towards this aim, it is also necessary to obtain a detailed description of the mechanisms involved in the Zn-Pc-photosensitised destruction of tumour tissues subjected to PDT. As a first step in this direction, we have performed ultrastructural studies on tumour samples obtained from mice bearing an MS-2 fibrosarcoma at various times after the PDT treatment in the presence of Zn-Pc. Previous investigations showed that this technique can provide useful information as regards the progress of tissue necrosis in tumours which have undergone PDT after administration of HpD (Bellnier & Lin, 1984; Zhou *et al.*, 1988*a*) and haematoporphyrin (Zhou *et al.*, 1988*b*).

### Materials and methods

The procedures adopted for the incorporation of Zn-Pc into small unilamellar liposomes of DPPC, its i.v. injection to Balb/c mice bearing a transplanted MS-2 fibrosarcoma in the right hind leg, and the irradiation of the tumour area with red light were the same as previously described (Reddi *et al.*, 1990).

In the experiments described in the present paper, the mice having a tumour with an external diameter equal to 0.6–0.7 cm received a Zn-Pc dose of 0.12 mg $kg^{-1}$ body weight. At 24 h after injection, the tumour area was depilated and exposed to 300 J $cm^{-2}$ of 590–740 nm light at a dose-rate of 180 mW $cm^{-2}$.

The mice were killed at 3, 6, 15, 24, 48 and 72 h after the end of the phototherapeutic treatment (three mice at each time) by exposure to vapours of diethyl ether. Small pieces of macroscopically non-necrotised tumour tissue were quickly removed. The specimens were fixed in 3% glutaraldehyde, 0.1 M cacodylate – buffered at pH 7.3, for 2 h at 4°C, post-fixed in 1% $OsO_4$ cacodylate – buffered for 1 h, dehydrated and embedded in Epon. The thin sections were doubly stained with uranyl acetate and lead citrate and then examined with a Philips EM-410 TEM and Hitachi H-600 TEM.

Control mice were represented by mice that were subjected to the above described PDT treatment in the absence of Zn-Pc. Previous studies (Milanesi *et al.*, 1987) had shown that the administration of liposome-bound Zn-Pc (0.5 mg $kg^{-1}$) causes no detectable ultrastructural alterations of the MS-2 fibrosarcoma in mice.

### Results

The tumours of unirradiated Balb/c mice were composed by densely arranged cells of polygonal or slightly elongated shape (Figure 1a). The nucleus is often rather large and contains a prominent nucleolus. The cytoplasm is characterised by the presence of somewhat scattered mitochondria with less frequent cristae, as is typical of several types of malignant cells (Pedersen, 1978), abundant free ribosomes, a few profiles of rough endoplasmic reticulum, and sometimes Golgi complex. The capillaries in the tumour tissue (Figure 1b) appear to be of continuous type and are composed by a very thin layer of endothelial cells. The ultrastructural features of tumours isolated from mice that had been irradiated in the absence of Zn-Pc are essentially identical with those observed for untreated mice. This suggests that irradiation alone exerts no tissue-damaging effects, at least under our experimental conditions.

At 3 h after PDT in the presence of liposome-delivered Zn-Pc the capillaries supplying the tumour tissue appear to be very well preserved (Figure 2a and b) being almost identical with those seen in the control mice. The congestion of the capillaries by erythrocytes may reflect an early dilation of vessels in the irradiated tumour (Star *et al.*, 1986). On the other hand, as one can observe in Figure 2, some malignant cells display some vacuolisation and markedly swollen and empty mitochondria, indicating that PDT induced an early degeneration of these compartments of malignant cells. The damage at the level of the malignant cells becomes even more evident at 6 h after irradiation (Figure 3): several tumour

Correspondence: G. Jari.
Received 7 September 1989; and in revised form 24 January 1990.

**Figure 1** **a**, Tumour cells of a control mouse are polygonal, with a large nucleus (N), mitochondria (m), abundant free ribosomes, profiles of rough endoplasmic reticulum (RER) and Golgi complex (G). × 9,000. **b**, Blood vessels in tumoral tissue are of continuous type. L = lumen; N = nucleus; E = endothelium. × 15,000.

**Figure 2** Typical micrographs obtained 3 h after PDT. **a**, The capillary supplying tumour cells is very well preserved; tumour cells contain some vacuolisation (v) and swollen mitochondria (arrows). × 9,000. **b**, Higher magnification shows more clearly that endothelial cells are much better preserved than neoplastic cells. × 17,000.

cells actually appear degenerative with largely swollen and sometime disrupted mitochondria and considerably dilated rough endoplasmic reticulum (Figure 3a).

Some individual cells are already necrotised and destroyed. On the contrary, the capillary endothelial cells (Figure 3b) continue to be well preserved, only minimal changes being observed in their cytoplasm. It is important to underline that closely similar ultrastructural data were obtained with different tissue specimens taken from different animal. Therefore, the results illustrated in Figures 1–3 (as well as in the subsequent figures) appear to be of general validity.

Only at 15 h after PDT (Figure 4) does the photoinduced necrotic process begin to involve the whole tumour tissue. The micrographs show a widespread occurrence of necrotised and disintegrated neoplastic cells, although some relatively more resistant cells are occasionally observed (see the tumour cell T adjacent to the erythrocytes in Figure 5). Moreover, some severely damaged capillaries with swollen or disrupted

**Figure 3** Typical micrographs obtained 6 h after PDT. **a**, Several tumour cells appear degenerative, with swollen mitochondria and dilated profiles of rough endoplasmic reticulum (arrows). ×12,000. **b**, Blood vessels are well preserved. N = endothelial nucleus; T = tumour cell. ×15,000.

**Figure 4** Fifteen hours after PDT, photoinduced damage involves both malignant and endothelial cells. Micrograph shows widespread degeneration of the tumoral tissue. E = endothelium; N = nucleus; RC = red blood cells. ×12,000.

mitochondria and a dilated endoplasmic reticulum are detected (Figure 4). At 24 h after irradiation, the tumour tissue is almost entirely necrotic and no viable-appearing tumour cells could be observed in any of the specimens taken up to 72 h. Occasionally, rather mildly damaged capillaries surrounded by completely necrotic tumour cells are detected even at 72 h after PDT (Figure 6).

In a few experiments, the tumour-bearing mice were i.v. injected with 0.12 mg $kg^{-1}$ Zn-Pc associated with human LDL. Upon irradiation of the tumour tissue we observed ultrastructural changes identical with those found in the case of mice that had received liposome-bound Zn-Pc.

**Figure 5** Tumour tissue is completely necrotic 24 h after PDT. T = tumour cell. ×8,500.

**Figure 6** A mildly damaged capillary, surrounded by completely necrotic tumour, is occasionally detected even 72 h after PDT. ×7,000.

### Discussion

Our ultrastructural studies with a PDT-treated experimental tumour further support the conclusion (Reddi *et al.*, 1990) that Zn-Pc is a very efficient phototherapeutic agent.

Extensive necrotic degeneration of the tumour tissues is obtained upon administration of 0.12 $mg\ kg^{-1}$ Zn-Pc, i.e. a dose at least 20-fold lower than that presently used for clinical PDT in the presence of HpD or Photofrin II (Dougherty, 1987). In particular, the electron microscopy data clearly indicate that the mechanism by which Zn-Pc induces the necrosis of irradiated tumours is remarkably different from that observed after PDT of tumours loaded with HpD (Henderson *et al.*, 1985; Star *et al.*, 1986; Chopp *et al.*, 1987) or water-soluble phthalocyanines, such as aluminum tetrasulphophthalocyanine (Selman *et al.*, 1986). With all these photosensitisers the tissue photodamage primarily involves the blood vessels, while the photodamage of the malignant cells either occurs at a slower rate or is an indirect consequence of the vascular damage. In contrast, for irradiations in the presence of Zn-Pc, the neoplastic cells clearly represent the initial direct target of PDT, and the capillaries in the tumour tissue are modified at later stage and to a less severe extent. Indeed, upon both gross inspection and microscopic examination, we found only low levels of haemorrhage in the necrotic tumours: haemorrhagic necrosis is very frequently observed during PDT with HpD.

We ascribe the different mechanism of tumour necrosis observed for PDT with Zn-Pc to the modality of transport and delivery of this drug to tumours. As discussed in a previous paper (Reddi *et al.*, 1990), Zn-Pc is selectively carried by serum lipoproteins, and hence it is likely that its release to malignant cells through receptor-mediated endocytosis of LDL plays an important role owing to the elevated number of LDL-receptors associated with neoplastic cells (Gal *et al.*, 1981).

This would explain the identity between the ultrastructural modifications detected after administration of liposome-bound or LDL-bound Zn-Pc. By this process, the photosensitising agent is specifically delivered to malignant cells and localises in the cellular membranes (Brown *et al.*, 1980; Candide *et al.*, 1986). On the other hand, more polar photosensitisers, which can be injected as a homogeneous aqueous solution, are largely transported by albumin and other serum proteins and localise mainly in the vascular stroma (Kessel *et al.*, 1987).

The proposed sequence of events is in agreement with the pattern of ultrastructural changes observed in our experimental tumour, namely the early alteration of the mitochondria and the cytoplasmic membrane of malignant cells. Moreover, we have previously shown (Zhou *et al.*, 1988*a*) that liposome- or LDL-delivered haematoporphyrin causes minimal degree of PDT-induced vascular damage and a direct photosensitisation of tumour cells, whereas the opposite situation takes place upon injection of aqueous haematoporphyrin. Therefore, it is likely that the mechanism of photoinduced tumour necrosis observed in the case of Zn-Pc is not a unique property of the dye, but is a consequence of the transport mechanism. Of course, more detailed experimental studies are to be performed in order to corroborate our hypothesis. In any case, the results described in this paper open two interesting prospects.

1. The possibility of achieving the specific delivery to tumour cells *in vivo* of any hydrophobic photosensitiser which can be incorporated into liposomal vesicles or LDL. This would yield a large flexibility in the choice of the phototherapeutic agent, whose spectroscopic and photosensitising properties could be tailored to the optical features and biochemical or physiological characteristics of the tumour to be irradiated. In fact, we have recently obtained a selective accumulation of other liposome-bound dyes, such as porphycenes and naphthalocyanines, by experimental tumours.

2. The continuation of oxygen supply to tumours for some hours after PDT. This circumstance may potentiate the PDT-induced damage which is known to be of oxidative nature (Lee See *et al.*, 1984) by reducing the hypoxic areas with only partially modified neoplastic cells (Freitas, 1985), similar to what is often observed to occur in the radiotherapy of tumours (Moulder & Rockwell, 1984). Such areas are more likely to be formed as a consequence of vascular damage and are a common origin of tumour recurrences.

### References

BELLNIER, D.A. & LIN, C.W. (1984). Giant cell formation in bladder tumor cells following hematoporphyrin derivative-sensitized photoirradiation. *Photochem. Photobiol.*, **39**, 425.

BROWN, M.S., KOVANEN, P.T. & GOLDSTEIN, J.L. (1980). Evolution of the LDL receptor concept from cultured animal cells to intact animals. *Proc. NY Acad. Sci.*, **348**, 48.

CANDIDE, C., MORLIERE, P., MAZIERE, J.C. & 5 others (1986). In vitro interaction of the photoactive anticancer porphyrin derivative photofrin II with low density lipoprotein and its delivery to cultered human fibroblasts. *FEBS Lett.*, **33**, 183.

CHOPP, M., GLASBERG, M.R., RIDDLE, J.M., HETZEL, F.W. & WELCH, K.M.A. (1987). Photodynamic therapy of normal cerebral tissue in the cat: a non-invasive model for cerebrovascular thrombosis. *Photochem. Photobiol.*, **46**, 103.

DOUGHERTY, T.J. (1987). Photosensitizers: therapy and detection of malignant tumors. *Photochem. Photobiol.*, **45**, 879.

FREITAS, I. (1985). Role of hypoxia in photodynamic therapy of tumors. *Tumori*, **71**, 251.

GAL, D., MCDONALD, P.C., PORTER, J.C. & SIMPSON, E.R. (1981). Cholesterol metabolism in cancer cells in monolayer culture. III. Low-density lipoprotein metabolism. *Int. J. Cancer*, **29**, 315.

HENDERSON, B.W., WALDOW, S.M. & MANG, T.S. (1985). Tumor destruction and kinetics of tumor cell death in two experimental mouse tumors following photodynamic therapy. *Cancer Res.*, **45**, 572.

JORI, G. (1987). Photodynamic therapy of solid tumors. *Radiat. Phys. Chem.*, **30**, 375.

KESSEL, D., THOMPSON, P., SOATIO, K. & NANTURI, K.D. (1987). Tumor localization and photosensitization by sulfonated derivatives of tetraphenylporphine. *Photochem. Photobiol.*, **45**, 787.

LEE SEE, K., FORBES, I.J. & BETTS, W.H. (1984). Oxygen dependency of phototoxicity with hematoporphyrin derivative. *Photochem. Photobiol.*, **39**, 631.

MILANESI, C., BIOLO, R., REDDI, E. & JORI, G. (1987). Ultrastructural studies on the mechanism of the photodynamic therapy of tumors. *Photochem. Photobiol.*, **46**, 675.

MOULDER, J. & ROCKWELL, S. (1984). Hypoxic fractions of solid tumors: experimental techniques, methods of analysis, and a survey of existing data. *Int. J. Radiat. Oncol. Biol. Phys.*, **10**, 695.

PEDERSEN, F.L. (1978). Tumor mitochondria and the bioenergetics of cancer cells. *Prog. Exp. Tumor Res.*, **22**, 190.

REDDI, E., ZHOU, C., BIOLO, R., MENEGALDO, E. & JORI, G. (1990). Zn(II)-phthalocyanine as a photodynamic agent for tumours. I. Pharmacokinetic properties and phototherapeutic efficiency. *Br. J. Cancer* (in the press).

SELMAN, S.H., KREIMER-BIRNBAUM, M., CHADHURI, K. & 5 others (1986). Photodynamic treatment of transplantable bladder tumors in rodents after pretreatment with chloroaluminum tetrasulfophthalocyanine. *J. Urol.*, **136**, 141.

STAR, W.M., MARIJNISSEN, H.P.A., VAN DEN BERG-BLOK, A.E., VERSTEEG, J.A., FRANKEN, K.A. & REINHOLD, H.S. (1986). Destruction of rat mammary tumor and normal tissue microcirculation by hematoporphyrin derivate. Photoradiation observed in sandwich observation chambers. *Cancer Res.*, **46**, 2532.

ZHOU, C.N., XU, B.J., XIE, J.G. & 6 others (1988*a*). An ultrastructural study of human bladder cancer treated with photodynamic therapy. *Lasers Med. Sci.*, **3**, 87.

ZHOU, C.N., MILANESI, C. & JORI, G. (1988*b*). An ultrastructural comparative evaluation of tumors photosensitized by porphyrins administered in aqueous solution, bound to liposomes or to lipoproteins. *Photochem. Photobiol.*, **48**, 487.